# AI-Facilitated Discovery of Microbiome-Originated Antimicrobial Peptides: A Perspective on Combating Antibiotic Resistance Within Public Health and Food Safety Frameworks

**DOI:** 10.3390/microorganisms14102360

**Published:** 2026-10-15

**Authors:** Paul Y. Atuahene, Bryanna Stevenson, Yongming Sang

**Affiliations:** Department of Food and Animal Sciences, College of Agriculture, Tennessee State University, Nashville, TN 37209, USA; patuahen@my.tnstate.edu (P.Y.A.); bsteve10@my.tnstate.edu (B.S.)

**Keywords:** antimicrobial peptides, artificial intelligence, microbiome, antibiotic resistance, machine learning, public health microbiology, food safety

## Abstract

The escalating crisis of antimicrobial resistance (AMR) demands innovative therapeutic strategies beyond conventional antibiotics. Antimicrobial peptides (AMPs), particularly those originating from the human, animal, and environmental microbiome, represent a promising frontier within public health microbiology and microbial food safety. Recent advances in artificial intelligence (AI) and machine learning (ML) have revolutionized the discovery pipeline, enabling high-throughput mining of metagenomic data and de novo generation of novel peptide sequences. This perspective review synthesizes recent breakthroughs in AI-driven discovery of microbiome-originated AMPs, examines their preclinical efficacy against multidrug-resistant (MDR) pathogens under One Health and climate-sensitive frameworks and application in infectious disease treatment and discusses translational challenges including pharmacokinetics, toxicity, manufacturing scalability, and applications in food preservation and water safety. We argue that the convergence of microbiome science, deep learning, and synthetic biology positions AI-discovered AMPs as viable next-generation interventions against antibiotic resistance across human, animal, and environmental domains.

## 1. Introduction

The discovery of penicillin by Alexander Fleming in 1928 marked the dawn of the antibiotic era, transforming infectious disease treatment and dramatically reducing mortality from bacterial infections [1]. However, the subsequent decades have witnessed the emergence and global spread of antimicrobial resistance (AMR), rendering many conventional antibiotics ineffective against previously susceptible pathogens. The World Health Organization (WHO) has declared AMR as one of the top ten global public health threats facing humanity, with recent estimates suggesting that bacterial AMR was associated with approximately 4.95 million deaths in 2019, including 1.27 million deaths directly attributable to resistant infections [2].

The economic and healthcare burden of AMR continues to escalate. Projections indicate that without intervention, AMR could cause 10 million deaths annually by 2050, with cumulative economic costs exceeding 100 trillion USD [3]. The pipeline for new antibiotic development has dwindled significantly, with only a handful of novel classes approved in recent decades. This crisis is further exacerbated by the emergence of multidrug-resistant (MDR) “superbugs,” including the ESKAPE pathogens (*Enterococcus faecium*, *Staphylococcus aureus*, *Klebsiella pneumoniae*, *Acinetobacter baumannii*, *Pseudomonas aeruginosa*, *and Enterobacter species*), which are responsible for the majority of nosocomial infections and have been designated as critical or high priority by the WHO [4,5].

In this challenging landscape, antimicrobial peptides (AMPs) have emerged as promising candidates for next-generation antimicrobial therapies. AMPs are evolutionarily conserved components of the innate immune system found across all domains of life, ranging from prokaryotes to mammals [6,7]. Unlike conventional antibiotics that typically target specific cellular processes, AMPs generally exert their antimicrobial effects through membrane disruption and other nonspecific mechanisms, making them less susceptible to the development of bacterial resistance [8]. Furthermore, many AMPs exhibit immunomodulatory properties and can disrupt biofilm formation and quorum sensing—critical virulence factors in persistent foodborne and healthcare-associated infections [9,10].

The human microbiome, along with its animal and environmental counterparts, harbors trillions of microorganisms and constitutes a vast, underexplored reservoir of bioactive molecules, including AMPs. Bacteriocins—ribosomally synthesized antimicrobial peptides produced by bacteria—are particularly abundant in the gut microbiota, where they serve competitive functions in microbial community dynamics [6,9]. Recent studies have demonstrated that microbiome-derived AMPs exhibit potent activity against clinically relevant pathogens while maintaining selectivity for bacterial over mammalian cells [10,11]. The environmental microbiome, including soil and water reservoirs, represents an equally important yet underexplored source of novel AMPs, with direct implications for water safety and environmental health within the One Health paradigm [11].

The integration of artificial intelligence (AI) and machine learning (ML) methods with microbiome research has revolutionized the pace and scale of AMP discovery. Traditional experimental screening approaches, while valuable, are time-consuming, expensive, and limited in throughput. In contrast, computational methods can screen millions of sequences in silico, predict functional properties, and even generate novel peptides with desired characteristics [12,13]. Deep learning architectures, including convolutional neural networks (CNNs), recurrent neural networks (RNNs), and transformer-based models, have demonstrated remarkable accuracy in predicting antimicrobial activity from peptide sequences [14,15]. This perspective review provides an updated examination of AI-facilitated discovery of microbiome-originated AMPs and their potential as interventions against antibiotic resistance within the context of public health microbiology, microbial food safety, and the One Health framework. We discuss the computational methodologies that underpin these advances, highlight recent landmark discoveries, address current challenges and limitations, and outline future directions for translating computational predictions into clinical and food safety applications.

## 2. Background: The Antibiotic Resistance Crisis and the Promise of AMPs

### 2.1. The Global Burden of Antimicrobial Resistance

Antimicrobial resistance (AMR) has transcended its origins as a localized clinical challenge to become one of the most formidable threats to global public health and modern medicine. The landmark 2019 Global Burden of Disease study, representing the most comprehensive assessment of this crisis to date, quantified its devastating toll: bacterial AMR was directly implicated as the cause of an estimated 1.27 million deaths and was associated with a staggering 4.95 million deaths across 204 countries and territories [2]. These figures place AMR as a leading cause of mortality worldwide, surpassing many single infectious diseases. The burden is disproportionately borne by low- and middle-income countries, where healthcare infrastructure and access to novel therapeutics are limited [16,17].

The mechanistic basis of antibiotic resistance is diverse and continually evolving. Bacteria have developed multiple strategies to evade antibiotic action, including enzymatic inactivation (e.g., beta-lactamases), target modification (e.g., altered penicillin-binding proteins), efflux pump upregulation, and reduced membrane permeability [18,19]. Horizontal gene transfer facilitates the rapid dissemination of resistance determinants among bacterial populations, including between commensal and pathogenic species, as well as across human, animal, and environmental reservoirs [20,21]. This interdomain transmission underscores the necessity of a One Health approach to AMR surveillance and intervention.

The clinical pipeline for new antibiotics has reached critically low levels. Between 2017 and 2021, only 11 new antibiotics were approved by the FDA, and the majority of these were derivatives of existing classes rather than novel chemical entities [22]. The pharmaceutical industry has largely withdrawn from antibiotic research due to poor economic returns, creating an innovation gap that urgently needs to be addressed through alternative approaches [22,23,24]. The 2024 WHO Bacterial Priority Pathogens List reinforces the urgency, with carbapenem-resistant *Enterobacterales*, *Acinetobacter baumannii*, and rifampicin-resistant *Mycobacterium tuberculosis* ranked as critical priorities, while fluoroquinolone-resistant *Salmonella* and *Shigella* spp. have risen in priority due to their foodborne transmission and disproportionate burden in resource-limited settings [24].

### 2.2. Antimicrobial Peptides as Alternative Therapeutics

AMPs offer several advantages over conventional antibiotics that make them attractive candidates for addressing the AMR crisis:

(1) Broad-spectrum activity: Many AMPs exhibit activity against Gram-positive and Gram-negative bacteria, fungi, and viruses, often through mechanisms that are less susceptible to resistance development [6,25,26,27].
(2) Rapid killing kinetics: AMPs typically kill bacteria within minutes through membrane disruption, compared to hours or days for many conventional antibiotics [28,29].
(3) Immunomodulatory effects: Beyond direct antimicrobial activity, many AMPs modulate immune responses, reducing inflammation and enhancing pathogen clearance [30,31].
(4) Low resistance potential: The nonspecific membrane-targeting mechanisms of many AMPs make it difficult for bacteria to develop resistance through single-point mutations [25,29,32].
(5) Anti-biofilm and anti-quorum sensing activity: Several microbiome-derived AMPs and bacteriocins can inhibit biofilm formation and disrupt quorum sensing networks, addressing persistent infections that evade conventional antibiotic therapy [9,33].

### 2.3. The Microbiome as a Reservoir of Novel AMPs

The human microbiome comprises approximately 10^14^ microorganisms, encoding over 100-fold more genes than the human genome [34,35]. This vast genetic repository includes numerous biosynthetic gene clusters (BGCs) encoding bioactive molecules, including AMPs and bacteriocins. The competitive environment of microbial communities has driven the evolution of diverse antimicrobial compounds that mediate interspecies competition and community structure [36,37].

Bacteriocins, in particular, represent a major class of microbiome-derived AMPs. These ribosomally synthesized peptides are produced by bacteria across diverse phyla and exhibit remarkable structural and functional diversity [6,9,38]. Class I bacteriocins undergo extensive post-translational modifications, while Class II bacteriocins are unmodified or minimally modified peptides [9]. The gut microbiota, in particular, harbors extensive bacteriocin-encoding potential, with recent studies identifying thousands of novel bacteriocin gene clusters through metagenomic analysis [38,39].

The animal and environmental microbiomes represent equally valuable reservoirs. The ruminant gastrointestinal microbiome, with its specialized fermentation metabolism and dense microbial communities, has yielded promising anti-MRSA candidates [40]. Environmental metagenomes from soil and aquatic ecosystems have expanded known AMP diversity by orders of magnitude, with many candidates showing potent activity against WHO priority pathogens [11]. These findings align with the One Health model, recognizing that microbial diversity and resistance dynamics are interconnected across human, animal, and environmental domains.

Despite this potential, the vast majority of microbiome-derived AMPs remain undiscovered. Traditional bioassay-guided fractionation approaches are limited by the unculturable nature of many microbiome constituents and the labor-intensive nature of experimental screening. Computational methods offer a scalable alternative for exploring this vast chemical space.

## 3. Artificial Intelligence and Machine Learning Approaches for AMP Discovery

### 3.1. Overview of Computational AMP Discovery

**Figure 1:**
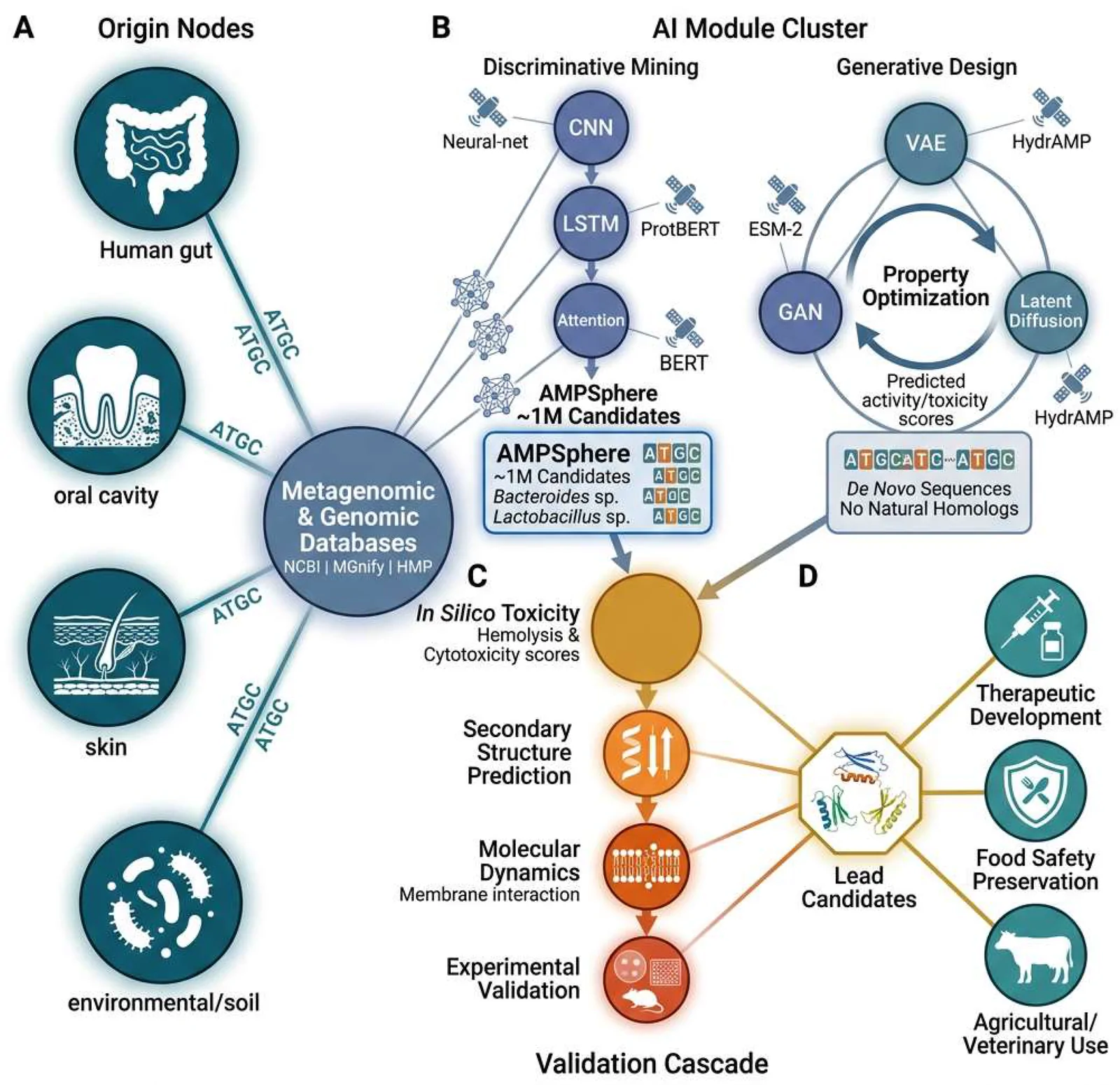
AI-driven discovery pipeline for microbiome-originated antimicrobial peptides. The pipeline comprises four integrated stages. (**A**) Data input layer: Metagenomic and genomic sequences from diverse microbiome sources (human gut, oral cavity, skin, and environmental/animal samples) are aggregated into public databases (NCBI, MGnify, Human Microbiome Project). (**B**) AI mining and generation modules: Two complementary computational approaches operate in parallel—(**left**) discriminative mining using deep learning architectures (CNN, LSTM, attention mechanisms, BERT/Transformer models) to extract candidate AMPs from genomic data, yielding large-scale repositories such as AMPSphere (~1 million candidates); and (**right**) generative design employing variational autoencoders (VAE), generative adversarial networks (GAN), and latent diffusion models to create de novo peptide sequences with optimized properties, guided by iterative feedback from activity and toxicity predictors. (**C**) Filtering and validation funnel: Candidate peptides undergo hierarchical down-selection through in silico toxicity prediction (hemolysis, cytotoxicity), secondary structure prediction (α-helix, β-sheet), molecular dynamics simulation of membrane interactions, and experimental validation (antimicrobial activity assays, MIC determination, in vivo efficacy models). (**D**) Output and applications: The funnel converges on 3–5 lead candidates, which are directed toward therapeutic development, food safety preservation, or agricultural/veterinary applications. Computational stages are depicted in cool tones (blues/teals), biological validation in warm tones (oranges/reds), and lead candidates in gold accents. Key AI tools (ESM-2, ProtBERT, HydrAMP) are indicated as inset icons. Diagram is generated using Biorender and further polished using Gamma Graphic design. Created in BioRender. Li, J. (2026) https://BioRender.com/4kfhn67.

The application of AI and ML to AMP discovery has evolved through several generations of methodological advances. Early approaches relied on sequence alignment and motif-based screening, while contemporary methods leverage sophisticated deep learning architectures capable of capturing complex sequence patterns and relationships [10,11,12,13,14,15]. The computational AMP discovery pipeline typically involves several key stages (Figure 1):

(1) Data collection and curation: Assembly of training datasets from public AMP databases, including experimentally validated sequences and functional annotations.
(2) Feature representation: Encoding of peptide sequences into numerical representations suitable for machine learning, ranging from simple physicochemical descriptors to learned embeddings.
(3) Model training: Development of predictive models using supervised, unsupervised, or self-supervised learning approaches.
(4) Virtual screening: Application of trained models to large sequence databases for candidate identification.
(5) Hit prioritization: Ranking of candidates based on predicted properties, including activity, toxicity, and stability.
(6) Experimental validation: Laboratory testing of prioritized candidates to confirm predicted activities (Figure 1).

### 3.2. Discriminative Models for AMP Classification

Discriminative models form the foundation of computational AMP discovery, learning to distinguish antimicrobial from non-antimicrobial sequences based on training data. These approaches have demonstrated high accuracy and have been widely deployed for genome and metagenome mining.

#### 3.2.1. Traditional Machine Learning Approaches

Traditional ML methods, including random forests, support vector machines (SVMs), and gradient boosting machines, have been successfully applied to AMP prediction [14,41,42,43]. These models typically rely on handcrafted features describing peptide physicochemical properties, including:

• Charge and isoelectric point.
• Hydrophobicity and amphipathicity.
• Amino acid composition and frequencies.
• Secondary structure propensities.
• Sequence-derived descriptors (e.g., k-mer frequencies).

Notable traditional ML tools include CAMP (Collection of Anti-Microbial Peptides) [43], iAMPpred [44], and ampir [41] (Table 1), which have achieved accuracies exceeding 90% on benchmark datasets. The ampir package, specifically designed for genome-wide screening, demonstrated significantly higher classification accuracy than existing methods when applied to whole-genome data [42].

#### 3.2.2. Deep Learning Architectures

Deep learning approaches have substantially advanced AMP prediction capabilities by automatically learning informative features from raw sequence data, eliminating the need for manual feature engineering.

Convolutional Neural Networks (CNNs): CNNs excel at capturing local sequence patterns and have been successfully applied to AMP classification. Models such as AMP Scanner [41] and Deep-AMP [14] employ CNN architectures with multiple convolutional layers followed by pooling and fully connected classification layers (Table 1). These models achieve high accuracy while providing interpretable attention maps highlighting sequence regions important for antimicrobial activity.

Recurrent Neural Networks (RNNs): RNNs, particularly Long Short-Term Memory (LSTM) networks, are well-suited for modeling sequential dependencies in peptide sequences. The AMPlify model employs an attention-based LSTM architecture that achieved state-of-the-art performance on multiple benchmark datasets [15] (Table 1). Wang et al. [47] developed an LSTM-based deep learning model for the discovery of AMPs targeting drug-resistant *Mycobacterium tuberculosis*.

Transformer Models: Transformer architectures, which have revolutionized natural language processing, have been increasingly adopted for biological sequence analysis. The self-attention mechanism enables transformers to capture long-range dependencies and contextual relationships within sequences. Recent studies have demonstrated that transformer-based models, including protein language models like ESM-2 and ProtTrans, can effectively predict AMP activity and other functional properties [48,49].

### 3.3. Generative Models for De Novo AMP Design

While discriminative models identify AMPs from existing sequences, generative models can create entirely novel peptides with desired properties. This capability is particularly valuable for expanding the chemical space beyond naturally occurring sequences.

#### 3.3.1. Variational Autoencoders (VAEs)

VAEs learn compressed latent representations of peptide sequences that can be sampled to generate novel candidates. PepVAE [50], a semi-supervised VAE framework, learns a disentangled antimicrobial attribute space that enables controllable generation of AMPs with specific properties. Recent advances have extended VAE approaches to incorporate structural information and multi-objective optimization [51].

#### 3.3.2. Generative Adversarial Networks (GANs)

GANs employ adversarial training between a generator network that produces synthetic sequences and a discriminator network that distinguishes real from generated sequences [45,52,53]. For examples, the dsAMPGAN model utilizes a GAN architecture to generate AMP candidates with physicochemical properties similar to natural antimicrobial peptides [45]. GAN-based approaches have demonstrated the ability to generate diverse, novel sequences with high predicted antimicrobial activity [45,52,53].

#### 3.3.3. Autoregressive Models and Protein Language Models

Large protein language models trained on massive sequence databases can generate novel protein and peptide sequences that capture the statistical patterns of natural proteins. These models, including ProGen2 [46], deepAMP [54] and ESM-3 [55], can be fine-tuned or prompted to generate AMP-like sequences. The ApexAmphion framework couples a 6.4-billion-parameter protein language model with reinforcement learning for de novo antibiotic design, achieving a 100% hit rate in experimental validation of 100 designed peptides [56,57] (Table 1).

### 3.4. Multi-Objective Optimization Frameworks

Therapeutic development requires optimization of multiple properties beyond antimicrobial activity, including toxicity, stability, and manufacturability. Multi-objective optimization frameworks integrate multiple predictive models to guide the design of peptides with balanced property profiles.

The MAC-AMP system [48] represents a recent advance in this direction, employing a closed-loop multi-agent collaboration framework based on large language models. This system autonomously optimizes AMP generation for antibacterial activity, AMP-likeness, toxicity compliance, and structural reliability. Similarly, reinforcement learning approaches have been applied to optimize peptide sequences against composite reward functions incorporating multiple objectives [54,56].

## 4. Landmark Discoveries of Microbiome-Originated AMPs

### 4.1. Global Microbiome Mining Efforts

Recent large-scale computational studies have demonstrated the immense potential of microbiome-derived AMPs. Ma et al. [10] developed a deep learning approach to identify AMPs from the human gut microbiome, screening over 2000 human gut metagenomes and identifying hundreds of novel AMP candidates. Experimental validation confirmed that 78 of these predicted AMPs exhibited antimicrobial activity, with several showing potent activity against MDR pathogens including MRSA and *A. baumannii* [10].

Santos-Junior et al. [11] conducted an even more expansive analysis, mining 63,410 metagenomes from diverse environments using machine learning approaches. This study identified 863,498 novel AMP sequences, constituting a vast expansion of known AMP diversity. Functional profiling revealed that these peptides exhibited activity spectra spanning antibacterial, antifungal, and antiviral properties. Notably, many of the most potent candidates originated from underexplored microbial taxa, highlighting the value of broad taxonomic sampling [11] (Table 2).

### 4.2. Bacteriocin Discovery from Commensal Bacteria

Lactic acid bacteria (LAB) and other commensal organisms have been particularly fruitful sources of novel bacteriocins. Du et al. [58] conducted a large-scale genomic and machine learning analysis of the *Lactobacillaceae* family, identifying over 1000 novel bacteriocin gene clusters. Experimental characterization of selected candidates revealed potent activity against clinically relevant pathogens with minimal toxicity to mammalian cells [58].

Shen et al. [40] focused on the ruminant gastrointestinal microbiome, employing deep learning to identify novel AMPs from this unique ecosystem. This study identified a promising anti-MRSA candidate, demonstrating that microbiomes from diverse host species can yield therapeutically relevant AMPs. The ruminant gut, with its specialized fermentation metabolism and dense microbial communities, represents a particularly rich source of novel antimicrobial compounds [40].

### 4.3. Activity Against WHO Priority Pathogens

A critical test of microbiome-derived AMPs is their activity against the WHO priority pathogens responsible for the majority of MDR infections. Several recent studies have demonstrated potent activity against these challenging organisms:

(1) *Acinetobacter* *baumannii*: Novel peptides identified from gut microbiome metagenomes showed potent activity against carbapenem-resistant *A. baumannii* strains, with minimum inhibitory concentrations (MICs) in the low micromolar range [10].
(2) *Pseudomonas aeruginosa*: Deep learning-guided discovery identified peptides active against MDR *P. aeruginosa*, including strains resistant to last-resort antibiotics [13].
(3) *Klebsiella pneumoniae*: Bacteriocins from commensal bacteria demonstrated selective activity against carbapenem-resistant K. pneumoniae while sparing beneficial gut microbiota members [58].
(4) *Staphylococcus aureus*: Multiple studies have identified peptides with potent anti-MRSA activity from diverse microbiome sources [40,47].
(5) *Salmonella enterica* and *Shigella* spp.: The 2024 WHO BPPL elevated fluoroquinolone-resistant *S. typhi* and *Shigella* to high priority due to their foodborne transmission and burden in LMICs. *Lactobacillaceae-*derived AMPs have shown promising activity against these enteric pathogens [24,58].

In addition to the aforementioned studies, Table 3 summarizes the activities of some selected AI-driven AMPs and their reported activities against pathogenic microorganisms.

### 4.4. Novel and Multi-Target Mechanisms of Action

**Figure 2:**
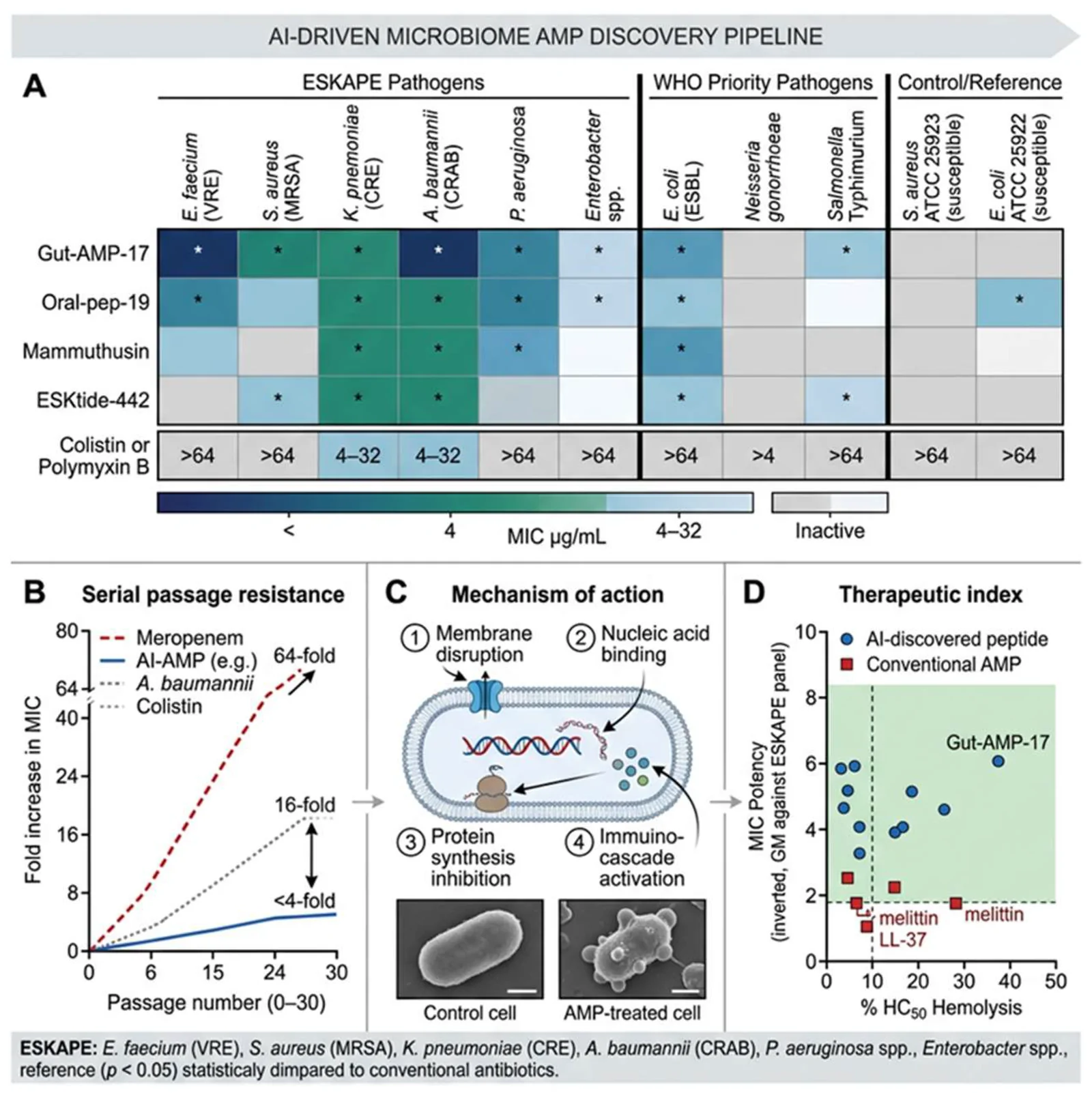
Activity profile and therapeutic potential of some AI-discovered microbiome AMPs against ESKAPE pathogens and clinical isolates. (**A**) Heatmap of antimicrobial activity: Minimum inhibitory concentrations (MICs, μg/mL) of AI-discovered peptides (rows) against ESKAPE pathogens, WHO priority pathogens, and susceptible reference strains. Color intensity indicates activity: dark blue (MIC < 4 μg/mL, highly active), light blue (4–32 μg/mL, moderate), white/gray (>64 μg/mL, inactive). A reference row shows colistin/polymyxin B MICs for comparison; asterisks denote statistically significant differences from conventional antibiotics (*p* < 0.05). (**B**) Resistance induction kinetics: Serial passage evolution of MIC fold-increase over 30 days for meropenem against K. pneumoniae (dashed red, rapid 64-fold increase by day 15), AI-AMP Gut-AMP-17 against K. pneumoniae (solid blue, <4-fold increase), and colistin against A. baumannii (dotted gray, 16-fold increase). (**C**) Mechanism of action summary: Multi-target activity of AI-discovered AMPs illustrated on a bacterial cell schematic, including (1) membrane disruption via pore formation, (2) nucleic acid condensation, (3) ribosome binding and protein synthesis inhibition, and (4) immunomodulatory cytokine activation. Insets show scanning electron micrographs of membrane blebbing in AI-AMP–treated A. baumannii versus intact control cells. (**D**) Therapeutic index comparison: Scatter plot of hemolytic activity (% HC_50_) versus geometric mean MIC against the ESKAPE panel for AI-discovered peptides (blue circles) and conventional AMPs (red squares; e.g., melittin, LL-37). The green-shaded quadrant denotes the ideal therapeutic window (low hemolysis, high potency); the vertical dashed line indicates the 10% hemolysis threshold. Diagram is generated using Biorender and further polished using Gamma Graphic design based on key references [60,61,62,63,64,65,66]. Created in BioRender. Li, J. (2026) https://BioRender.com/4kfhn67.

Beyond canonical membrane disruption, AI-driven discovery has revealed microbiome-derived AMPs with novel and multifaceted mechanisms. Many computationally designed peptides engage multiple bacterial targets simultaneously, reducing the likelihood of resistance emergence and broadening therapeutic potential (Figure 2).

**Membrane disruption via pore formation.** The canonical AMP mechanism—disruption of bacterial membrane integrity through pore formation—is frequently retained among AI-discovered microbiome AMPs. Computational design has further optimized membrane-interacting properties beyond those typically observed in natural peptides. Liu et al. [67] reported deep learning-guided design of self-assembling AMPs that form nanofibrous structures on bacterial membranes, causing membrane disruption and cell death, with efficacy in mouse intestinal infection models and without inducing acquired drug resistance [67]. Scanning electron microscopy supports these predictions: AI-AMP-treated *Acinetobacter baumannii cells* display pronounced membrane blebbing and surface irregularities compared with intact controls, consistent with pore formation and loss of membrane integrity. This morphological evidence corroborates membrane-targeting mechanisms predicted computationally and validated through molecular dynamics simulations [62,63].

**Nucleic acid condensation.** Several AI-discovered AMPs penetrate the bacterial membrane and bind intracellular DNA or RNA, disrupting replication, transcription, and translation. Molecular de-extinction studies, which use deep learning to mine proteomes of extinct organisms, identified peptides targeting the bacterial cytoplasmic membrane rather than the outer membrane—a mechanism distinct from most known AMPs—with potent in vivo efficacy in mouse infection models [13]. Nucleic acid condensation appears particularly prevalent among AI-designed peptides with cationic residues optimized for intracellular penetration. Molecular dynamics simulations indicate that these peptides traverse the membrane bilayer and interact electrostatically with nucleic acids, leading to DNA compaction and functional inactivation [60,62]. This dual-targeting capability—membrane disruption followed by nucleic acid binding—offers a significant advantage over conventional antibiotics that typically engage a single cellular target.

**Ribosome binding and protein synthesis inhibition.** Recent computational and experimental studies have identified AI-discovered AMPs that inhibit bacterial protein synthesis through ribosome binding. Unlike traditional ribosome-targeting antibiotics such as macrolides or tetracyclines, these peptides appear to engage multiple ribosomal components simultaneously, reducing the probability of resistance-conferring mutations. AMP-2, AMP-11, AMP-17, AMP-24, and AMP-25, identified through latent diffusion modeling and molecular dynamics screening, showed in vitro activity against Pseudomonas aeruginosa, Acinetobacter baumannii, Escherichia coli, and Klebsiella pneumoniae; AMP-24 had a reported MIC of 6.25 μM against *A. baumannii* and *E. coli* and efficacy in a mouse lung-infection model [62]. Integrating ribosome binding with membrane disruption creates a “dual-hit” mechanism that markedly reduces resistant mutants in serial passage experiments. As shown in Figure 2B, AI-AMP Gut-AMP-17 exhibited less than a 4-fold MIC increase over 30 days of serial passage against K. pneumoniae, compared with a 64-fold increase for meropenem and a 16-fold increase for colistin against *A. baumannii.*

**Immunomodulatory cytokine activation.** Many AI-discovered AMPs retain or have been optimized for immunomodulatory functions, including activation of cytokine responses that enhance pathogen clearance (Figure 2C). Computational models can now predict immunomodulatory potential from sequence features, enabling design of peptides that combine direct antimicrobial activity with beneficial host-directed effects [30,31,48]. This dual functionality—direct killing plus immune enhancement—is particularly attractive for infections complicated by dysregulated inflammation.

**Integrated multi-target activity.** The convergence of membrane disruption, nucleic acid condensation, ribosome binding, and immunomodulatory activation within single AI-designed peptides represents a paradigm shift in antimicrobial design (Figure 2C). Computational approaches enable rational integration of structural features promoting each mechanism, creating peptides with unprecedented functional complexity. As illustrated in the therapeutic index comparison (Figure 2D), AI-discovered peptides such as Gut-AMP-17 occupy an ideal therapeutic window characterized by low hemolytic activity and high potency against ESKAPE pathogens—a profile difficult to achieve with conventional AMPs that often sacrifice selectivity for activity [60,62,65]. These multi-target mechanisms also align with the One Health framework by potentially reducing selection pressure for resistance across human, animal, and environmental reservoirs. In summary, AI-facilitated discovery has expanded the mechanistic repertoire of microbiome-derived AMPs beyond simple membrane disruption, providing a multi-pronged strategy that enhances potency, reduces resistance potential, and broadens therapeutic applicability.

## 5. AMPs in the Context of Public Health Microbiology and Food Safety

### 5.1. The One Health Paradigm

The One Health framework recognizes the interconnectedness of human, animal, and environmental health, particularly in the context of AMR. Microbiome-derived AMPs occupy a unique position within this paradigm, as their discovery and application span all three domains. The horizontal transfer of resistance genes between commensal and pathogenic bacteria, and across human–animal–environmental interfaces, necessitates interventions that are effective and sustainable across ecosystems [19,20,21].

AI-driven AMP discovery platforms can mine metagenomic data from diverse One Health reservoirs—human gut, livestock microbiomes, soil, and aquatic environments—to identify peptides with broad applicability. For example, the same computational pipelines used to identify human gut-derived AMPs can be applied to livestock microbiomes, reducing antibiotic use in agriculture and mitigating resistance selection pressure in food production systems [25,26]. This cross-domain applicability is a distinctive strength of AI-facilitated discovery compared to traditional antibiotic development.

### 5.2. Microbial Food Safety Applications

Foodborne pathogens, including MDR *Salmonella*, *Campylobacter*, *Listeria monocytogenes*, and *Shigella*, represent a major public health burden and are increasingly prioritized in the WHO BPPL 2024 [24]. Microbiome-derived AMPs and bacteriocins offer promising solutions for food preservation and pathogen control across the farm-to-fork continuum:

(1) **Natural food preservatives:** Bacteriocins such as nisin and pediocin have long been used as natural preservatives in dairy and meat products. AI-discovered novel bacteriocins with enhanced activity spectra could expand this application to ready-to-eat foods and minimally processed products where chemical preservatives are undesirable [9,37].
(2) **Livestock microbiome engineering:** Identifying and administering protective AMP-producing probiotic strains to livestock can reduce pathogen carriage (e.g., *Salmonella* in poultry, *E. coli* O157:H7 in cattle) before animals enter the food chain, representing a pre-harvest intervention strategy [26,27].
(3) **Post-harvest decontamination:** AMP-based sprays or coatings for fresh produce and meat surfaces can reduce pathogen loads without contributing to chemical residue concerns [68].

The *Lactobacillaceae*-derived AMPs identified by Du et al. [58] exemplify this potential, showing activity against *L. monocytogenes*, *S. enterica*, and *Vibrio parahaemolyticus*—pathogens central to seafood and dairy safety—while maintaining low cytotoxicity profiles.

### 5.3. Water Safety and Environmental Health

Waterborne transmission of resistant pathogens is an underappreciated driver of AMR dissemination, particularly in resource-limited settings where sanitation infrastructure is inadequate. The 2024 WHO BPPL explicitly highlights the need for water, sanitation, and hygiene (WASH) interventions to control community-acquired resistant infections [24].

Environmental microbiomes from aquatic ecosystems have yielded diverse AMP candidates with activity against priority pathogens [11]. These peptides could be engineered for water treatment applications, including point-of-use disinfection and biofilm control in distribution systems. The low resistance potential of AMPs makes them particularly attractive for environmental applications where sublethal antibiotic exposure currently drives resistance selection [20,21].

### 5.4. Climate-Sensitive Infectious Diseases

Climate change is altering the epidemiology of infectious diseases by expanding vector habitats, accelerating pathogen evolution, and increasing the frequency of extreme weather events that disrupt sanitation and food systems. Rising temperatures and altered precipitation patterns have been linked to increased incidence of foodborne illnesses, including *Salmonella* and *Vibrio* infections, as well as expanded ranges for vector-borne pathogens [69,70].

AMPs offer climate-resilient therapeutic options for several reasons. First, their broad-spectrum activity is less dependent on precise strain identification, which is advantageous when surveillance systems are disrupted by climate events. Second, AI-driven discovery can rapidly pivot to address emerging pathogens as climate shifts alter disease burdens. Third, microbiome-derived AMPs that function across diverse environmental conditions (temperature, pH, salinity) can be prioritized for applications in climate-vulnerable regions where cold chain-dependent biologics are impractical.

## 6. Challenges and Limitations

### 6.1. Data Quality and Standardization

The foundation of any AI-driven discovery pipeline is the quality, quantity, and standardization of training data. Despite the exponential growth of AMP databases and metagenomic repositories, significant challenges remain that directly impact the reliability and reproducibility of computational predictions.

Public AMP databases, including CAMP, APD3, and DBAASP, contain heterogeneous annotations derived from diverse experimental protocols. Minimum inhibitory concentrations (MICs), for instance, are determined under varying conditions of pH, ionic strength, medium composition, and inoculum size, introducing substantial variability that complicates cross-study comparisons and model training [43,44]. Furthermore, many databases contain redundant sequences, conflicting activity annotations, and incomplete metadata regarding target organisms, making it difficult to establish ground truth for model development.

The scarcity of high-quality negative data—sequences experimentally confirmed to lack antimicrobial activity—represents a particular challenge for discriminative model training. Most databases are enriched for positive examples, leading to class imbalance that can inflate apparent model performance while reducing real-world predictive accuracy [14,49]. Recent efforts, including the ESCAPE benchmark dataset, aim to address these issues by providing standardized, curated datasets with consistent activity measurements and clear train–test splits [49]. However, the adoption of such benchmarks remains limited, and the field lacks consensus on gold-standard evaluation metrics.

For microbiome-originated AMP discovery specifically, additional data challenges arise from the unculturable nature of many microbial taxa. Metagenomic sequences from these organisms may be fragmented, incompletely assembled, or contain sequencing errors that propagate through downstream prediction pipelines. The functional annotation of biosynthetic gene clusters (BGCs) encoding AMPs is further complicated by the diversity of post-translational modifications that are not evident from genomic sequence alone [33,58]. These limitations underscore the need for integrated approaches that combine genomic, transcriptomic, and metabolomic data to improve training data quality.

### 6.2. Model Generalizability and Interpretability

A critical limitation of current AI models for AMP discovery is their generalizability beyond the training distribution. Models trained on sequences from well-characterized organisms may fail to recognize AMPs from underexplored taxonomic groups, precisely those that are most likely to harbor novel chemistry [11]. This “domain shift” problem is particularly acute for microbiome-derived AMPs, where the vast majority of sequences come from uncultured organisms with no experimentally validated peptides.

Deep learning models, including CNNs, RNNs, and transformer architectures, are often described as “black boxes” because their internal decision-making processes are not readily interpretable. While attention mechanisms can highlight sequence regions important for prediction, translating these attention weights into mechanistic understanding of antimicrobial activity remains challenging [15,48]. For regulatory acceptance and rational peptide optimization, interpretable models that can explain why a given sequence is predicted to be active—and how modifications might alter activity—are essential.

Overfitting to training data represents a persistent risk, particularly when models are trained on relatively small datasets of experimentally validated AMPs. Rigorous cross-validation, external validation on independent datasets, and prospective experimental testing are necessary to establish model reliability [49]. However, the disconnect between in silico predictions and in vitro activity remains substantial; even the most sophisticated models achieve only moderate correlation between predicted and experimentally determined MIC values.

The challenge of predicting in vivo efficacy from in vitro data represents another critical gap. AI models are typically trained on in vitro activity data (e.g., MIC against planktonic bacteria), but therapeutic success depends on pharmacokinetic properties, stability in physiological fluids, immune interactions, and efficacy in animal infection models—all of which are poorly captured by current computational approaches [54,56]. Bridging this gap will require models trained on integrated datasets that include in vivo efficacy endpoints.

### 6.3. Experimental Validation Bottlenecks

The translation of computational predictions into experimentally validated AMPs remains a major bottleneck in the discovery pipeline. While in silico screening can evaluate millions of candidate sequences in hours, laboratory synthesis and testing of even hundreds of peptides requires substantial time, resources, and specialized infrastructure. This asymmetry between computational and experimental throughput limits the practical utility of AI-driven discovery.

High-throughput experimental platforms, including microfluidic systems, peptide microarrays, and automated liquid handling robots, offer potential solutions by enabling parallel testing of hundreds to thousands of candidates [63,64]. However, these platforms are not widely accessible, and their integration with computational pipelines remains technically challenging. The development of standardized protocols for peptide synthesis, purification, and activity testing is essential for reproducible and comparable results across laboratories.

The gap between in silico predictions and experimental validation is exemplified by the observation that even top-ranked candidates often fail to show activity when tested. This “false positive” rate reflects both model limitations and the inherent complexity of antimicrobial activity, which depends not only on sequence but also on peptide concentration, target membrane composition, and environmental conditions. Iterative feedback between computational prediction and experimental testing—the “design–build–test–learn” cycle—is essential for improving model accuracy and identifying truly promising candidates [52,56].

### 6.4. Integration with Experimental Workflows

The integration of AI models with automated experimental platforms represents a promising frontier for accelerating AMP discovery, but significant challenges remain. Closed-loop systems, in which AI-designed peptides are synthesized and tested by robotic platforms, with results fed back to improve the models, have demonstrated success in other areas of bioengineering but are only beginning to be applied to AMP discovery [52,56].

Technical challenges include the development of robust interfaces between computational models and experimental hardware, the automation of data analysis and quality control, and the management of large, heterogeneous datasets generated by high-throughput experiments. Additionally, the time required for peptide synthesis and testing—typically days to weeks per cycle—limits the speed at which models can be iteratively improved.

Interdisciplinary collaboration between computational and experimental researchers is essential for overcoming these barriers. Successful closed-loop systems require not only expertise in machine learning and bioinformatics but also in peptide chemistry, microbiology, and automation engineering. The establishment of shared platforms and standardized protocols for data exchange will facilitate broader adoption of these approaches.

### 6.5. Translational Challenges Specific to AI-Discovered AMPs

Beyond the general challenges of peptide therapeutics, AI-discovered AMPs present unique translational challenges that stem from their computational origins. The ability to predict pharmacokinetic properties—including absorption, distribution, metabolism, and excretion (ADME)—from sequence data remains limited. Current models focus primarily on antimicrobial activity and toxicity, with comparatively little attention to stability in physiological fluids, tissue penetration, or immunogenicity [71,72].

Toxicity prediction represents a critical area where AI can contribute, but also where model limitations have direct therapeutic implications. Hemolysis and cytotoxicity are among the most common reasons for failure of AMP candidates, and computational tools such as Hemolytik and ToxinPred provide valuable screening capabilities [71]. However, these models are trained primarily on data from natural AMPs and may not generalize to AI-generated sequences with non-natural modifications. Furthermore, predicting immunogenicity—the potential for peptides to elicit adverse immune responses—remains a largely unmet challenge.

Multi-objective optimization, in which peptides are designed to simultaneously satisfy multiple property requirements (activity, stability, safety, manufacturability), represents a promising approach but requires integration of multiple predictive models with potentially conflicting objectives [48,56]. The MAC-AMP system and similar frameworks have demonstrated the feasibility of this approach, but their practical utility depends on the accuracy of the underlying property predictors [48].

Regulatory considerations for AI-discovered therapeutics are still evolving. Agencies including the FDA and EMA have begun to develop frameworks for evaluating AI-based drug discovery and development, but clear guidance on data requirements, model validation, and documentation standards is lacking [73]. For AMPs specifically, the regulatory pathway may be further complicated by the novelty of mechanisms of action and the potential for broad-spectrum activity that affects commensal microbiota.

### 6.6. Intersection of AI and AMP-Specific Limitations

While the challenges described above primarily relate to AI methods, they intersect with inherent limitations of AMPs as therapeutic agents. Peptide stability, for instance, is both a biological challenge (proteolytic degradation) and a computational challenge (predicting stability from sequence). AI models can guide the design of stabilized peptides through incorporation of non-natural amino acids, cyclization, or PEGylation, but predicting the impact of these modifications on activity, safety, and manufacturability requires integrated models that capture complex structure–activity relationships [51,74].

Resistance development, while generally lower for AMPs than conventional antibiotics, remains a concern that AI can help address. Computational models can predict the propensity for resistance emergence based on mechanism of action, and generative approaches can design peptides with multiple targets that reduce the likelihood of resistance [56,75]. However, the evolutionary dynamics of resistance to AMPs are complex and not fully captured by current models.

Manufacturing scalability represents another area where AI can contribute but cannot fully solve inherent challenges. Peptide synthesis costs, particularly for sequences with non-natural amino acids or complex modifications, remain high. AI-guided optimization of production strains and bioprocess conditions can improve yields, but economic and logistical barriers to large-scale production persist [58,74].

In summary, the challenges facing AI-driven AMP discovery are multifaceted, spanning data quality, model generalizability, experimental validation, and translation to clinical and industrial applications. Addressing these challenges will require coordinated efforts across computational, experimental, and translational disciplines, with a particular emphasis on developing interpretable, generalizable models that can reliably predict in vivo efficacy and safety.

## 7. Future Directions

### 7.1. Integration of Large Language Models

The emergence of large language models (LLMs) for biological sequences promises to transform AMP discovery. Models trained on billions of protein sequences capture evolutionary relationships and functional constraints that enable accurate prediction of diverse protein properties. The application of LLMs to AMP discovery, including fine-tuning for antimicrobial activity prediction and peptide generation, represents a rapidly advancing frontier [48].

### 7.2. Integration with Microbiome Science

The microbiome is not merely a source of AMPs but a complex ecosystem where peptides function in context. Future AI models should integrate metagenomic, metabolomic, and transcriptomic data to understand how AMPs function within microbial communities. This systems-level approach could reveal synergistic peptide combinations and community-level effects that single-peptide assays miss.

### 7.3. Closed-Loop Experimental Platforms

The integration of computational design with automated experimental platforms enables closed-loop discovery workflows. In these systems, AI models design candidates, robotic platforms synthesize and test them, and results feed back to improve the models. Torres et al. [52] demonstrated this principle through generative Bayesian optimization, where peptide designs are iteratively refined based on experimental feedback. Such platforms dramatically accelerate the design–build–test–learn cycle and have demonstrated success in multiple areas of bioengineering [52,56].

### 7.4. Personalized Microbiome Therapeutics

The composition of the human microbiome varies significantly between individuals, influenced by diet, genetics, and environmental factors. Personalized approaches that identify AMPs from an individual’s own microbiome or design peptides matched to specific microbiome profiles may enhance therapeutic efficacy while minimizing disruption of beneficial commensals. AI models capable of predicting microbiome composition and host–pathogen–microbiome interactions will be essential for developing precision antimicrobial therapies [54,74].

### 7.5. Combination Therapies and One Health Applications

Combining AMPs with conventional antibiotics or other antimicrobial agents offers several potential advantages, including synergistic activity, reduced resistance development, and expanded spectrum. AI approaches for predicting drug interactions and optimizing combination regimens are being developed and may guide the rational design of AMP-based combination therapies [72]. Given the Special Issue’s focus on public health microbiology and microbial food safety, microbiome-derived AMPs have significant potential in food preservation and veterinary applications. The same AI pipelines used for human therapeutics can be applied to livestock microbiomes, reducing antibiotic use in agriculture and mitigating resistance selection pressure in food production systems [25,26].

### 7.6. Regulatory and Manufacturing Considerations

The clinical and food industry translation of AMPs requires navigating regulatory pathways and addressing manufacturing challenges. Unlike small-molecule antibiotics, peptides present unique manufacturing considerations, including synthesis complexity, stability during storage, and formulation requirements. Advances in continuous manufacturing, biosynthetic production, and synthetic biology may address these challenges and enable cost-effective production of therapeutic and food-grade AMPs (Table 4).

## 8. Conclusions

The convergence of microbiome science and artificial intelligence represents a paradigm shift in antimicrobial discovery with profound implications for public health microbiology and microbial food safety. AI-driven approaches have dramatically accelerated the identification of novel AMPs from microbiome sources, expanding known sequence diversity by orders of magnitude and revealing candidates with potent activity against the most challenging MDR pathogens. The integration of deep learning with large-scale metagenomic mining has transformed what was once a slow, serendipitous process into a systematic, high-throughput endeavor.

Recent successes, including the discovery of thousands of novel microbiome-derived AMPs with experimental validation of potent antimicrobial activity, demonstrate the power of these approaches. The ability to generate entirely novel peptides de novo, optimized for activity, stability, and safety, opens new dimensions in antimicrobial design that extend beyond natural chemical diversity.

However, significant challenges remain. The translation of computational predictions into clinically and industrially viable therapeutics requires addressing peptide stability, delivery, toxicity, and manufacturing considerations. The development of high-throughput experimental validation platforms and the integration of closed-loop AI-experimental systems will be critical for bridging the gap between in silico predictions and real-world applications.

Within the One Health framework, AI-facilitated AMP discovery offers a uniquely cross-cutting solution. The same computational pipelines can mine human, animal, and environmental microbiomes to identify peptides effective against priority pathogens while minimizing ecological disruption. Applications in food preservation, livestock protection, and water safety align with the Special Issue’s focus on public health microbiology and offer tangible pathways for reducing AMR selection pressure across the food chain.

Looking forward, the continued advancement of AI methods, including large language models and multi-agent systems, promises to further enhance discovery capabilities. The vision of personalized microbiome-derived antimicrobial therapies, tailored to individual patients and their specific infections, is moving closer to reality. In an era of climate-sensitive infectious diseases and expanding AMR, AI-discovered AMPs represent not merely an alternative to failing antibiotics, but a fundamentally new approach to safeguarding human, animal, and environmental health.

The global antibiotic resistance crisis demands urgent action and innovative solutions. AI-facilitated discovery of microbiome-originated AMPs offers a powerful approach for replenishing the antimicrobial pipeline with novel candidates that can address the most pressing clinical and food safety needs. By harnessing the vast chemical diversity encoded in the microbiome through the power of artificial intelligence, we can advance toward a future where effective treatments for MDR infections are readily available, safeguarding public health for generations to come.

## Figures and Tables

**Table 1:** Examples of AI tools, their application and reported output and website link.

Tool or Approach	Application	Reported Output	Link to Tool
**CAMP**	Database of AMP sequences and structures; prediction and benchmarking	Catalogued AMP sequences and structural records [43]	https://camp3.bicnirrh.res.in accessed on 28 September 2026
**iAMPpred**	AMP prediction from compositional, physicochemical, and structural features	Predicted AMP activity from peptide sequences [44]	https://dbaasp.org/tools?page=property-calculation accessed on 28 September 2026
**ampir**	Rapid genome-wide AMP screening with an R package	Putative AMPs from genomic sequences [41]	https://ampir.marine-omics.net accessed on 28 September 2026
**AMP Scanner**	Deep-learning recognition of AMPs from peptide sequence	Sequences classified as putative AMPs [42]	https://www.dveltri.com/ascan/ accessed on 28 September 2026
**Deep-AmPEP30**	Convolutional-network prediction of short AMPs (5–30 residues)	Short AMP candidates, including genome-screened leads [14,45]	https://app.cbbio.online/ampep/home accessed on 28 September 2026
**AMPlify**	Attention-based deep learning for AMP discovery	Novel candidates active against WHO priority pathogens [15]	https://bio.tools/amplify accessed on 28 September 2026
**ProGen2**	Protein language model for sequence generation	General protein sequences; potential design backbone [46]	https://bio-pro.mintlify.app/tools/gene-annotation/overview accessed on 28 September 2026

**Table 2:** Examples of Major AI-Driven Microbiome AMP Discovery Studies (2022–2025).

Study	Approach	Source	Key Findings
Ma et al., 2022 [10]	Deep learning (CAMP)	Human gut metagenomes (2000+)	78 experimentally validated AMPs; activity against MRSA
Santos-Junior et al., 2024 [11]	Machine learning ensemble	Global microbiome (63,410 metagenomes)	863,498 novel AMP sequences
Du et al., 2025 [58]	Genomics + ML	Lactobacillaceae family	1000+ novel bacteriocin clusters
Shen et al., 2025 [40]	Deep learning	Ruminant gut microbiome	Anti-MRSA candidate identified
Wan et al., 2024 [13]	Deep learning ensemble	Extinct organism proteomes	11,035 novel AMPs not found in extant organisms

**Table 3:** Some AI-designed antimicrobial peptides with reported activity against WHO-priority pathogens.

Peptide(s)	AI Approach	Pathogens Tested	Reported Experimental Evidence	Ref.
**pep-19-mod**	Explainable deep learning and virtual evolution	Carbapenem-resistant *E. coli*, *K. pneumoniae*, and *A. baumannii*; vancomycin-resistant *E. faecium*	Activity against the tested resistant strains; >95% reduction in bacterial burden in a mouse thigh-infection model.	[59]
**YI12; FK13**	Controllable deep generative model followed by molecular dynamics	MDR and polymyxin-resistant *K. pneumoniae*; *P. aeruginosa*; *A. baumannii*	Broad-spectrum in vitro activity with comparable MICs; low propensity for resistance in the reported experiments.	[51]
**PT-1, PT-4, PT-12, PT-15, PT-18, PT-20**	ProteoGPT protein-language-model pipeline	Carbapenem-resistant *A. baumannii* (CRAB); methicillin-resistant *S. aureus* (MRSA)	Six peptides showed broad-spectrum activity; MICs as low as 1 µg/mL. PT-1 and PT-18 had MRSA MICs of 4–13.3 µg/mL.	[60]
**m_AMP76; g_AMP41; g_AMP33; g_AMP35**	Protein language model, sequence mining, and generation	CRAB; MRSA	m_AMP76 MIC: 4 µg/mL against CRAB; g_AMP41 MIC: 8 µg/mL against MRSA. Seven leads entered mouse studies; in vitro potency did not always predict in vivo efficacy.	[60]
**AMP_S13**	BroadAMP-GPT generative and screening framework	MDR ESKAPE pathogens; MRSA in animal models	In vitro activity across an ESKAPE panel; improved survival in *Galleria* and wound healing in a mouse MRSA skin-infection model.	[61]
**AMP-2, AMP-11, AMP-17, AMP-24, AMP-25**	Latent diffusion model with molecular-dynamics screening	*P. aeruginosa*; *A. baumannii*; *E. coli*; *K. pneumoniae*	Selected peptides showed in vitro activity; AMP-24 had a reported MIC of 6.25 µM against *A. baumannii* and *E. coli* and efficacy in a mouse lung-infection model.	[62]
**GDST-038; GDST-045**	CalcAMP machine-learning screening	MDR ESKAPE strains; *A. baumannii*; *S. aureus*	Bactericidal activity and efficacy in a three-dimensional human skin infection model. GDST-038 showed no MIC increase against *A. baumannii* over 22 passages.	[63]
**Three leading hexapeptides**	Machine-learning pipeline mining hexapeptide sequence space	MDR clinical bacterial isolates	Broad activity against the tested isolates; aerosolized peptides showed efficacy comparable to penicillin in a mouse pneumonia model.	[64]
**Eight short AMPs**	Recurrent-neural-network generation with activity and hemolysis classifiers	*P. aeruginosa*; *A. baumannii*; MRSA	Eight synthesized peptides combined antimicrobial activity with low hemolysis in the reported in vitro assays.	[65]
**P001; P002; P076**	Generative adversarial network with activity prediction	MDR *A. baumannii*	Reported MICs against the resistant strain were 0.40, 0.47, and 0.21 µM, respectively; P076 showed lower hemolysis than P001/P002.	[66]

**Table 4:** Comparison between traditional or natural and AI-discovered AMP examples.

Feature	Traditional or Natural AMPs	AI-Discovered or Generated AMPs
**Bacterial potency**	Some natural AMP leads have limited activity or high minimum inhibitory concentrations (MICs) [76].	Selected optimized peptides achieved lower MICs than their study-specific comparators; one reported 10.6–64-fold higher potency than its parent peptide [54,77].
**Activity against resistant ESKAPE pathogens**	Proteolytic instability can reduce the activity of unmodified peptides [78].	Selected AI-discovered peptides showed activity against carbapenem-resistant Gram-negative bacteria and vancomycin-resistant *Enterococcus faecium* in experimental assays [59,79].
**Antifungal activity**	Antifungal activity varies among natural AMP candidates [80,81].	Some model-generated peptides showed activity against *Candida albicans* or *Candida glabrata*; this is model- and peptide-specific [60,62].
**In vivo efficacy**	Toxicity, degradation, and limited stability remain translational barriers for many AMP candidates [78,80].	Selected candidates reduced bacterial burden in mouse infection models, including a reported >95% reduction for one peptide; these are preclinical outcomes [11,59].
**Safety and selectivity**	Hemolysis and toxicity toward mammalian cells can restrict therapeutic use [82].	Multivariable screening can prioritize peptides with lower predicted or measured hemolysis and cytotoxicity, although each candidate still requires experimental testing [13,60,83].

## Data Availability

The original contributions presented in this study are included in the article. Further inquiries can be directed to the corresponding author.
